# Age-related neurodegenerative disease research needs aging models

**DOI:** 10.3389/fnagi.2015.00168

**Published:** 2015-09-02

**Authors:** Ian P. Johnson

**Affiliations:** Discipline of Anatomy and Pathology, Motoneurone Research Laboratory, The University of AdelaideAdelaide, SA, Australia

**Keywords:** aging, development, neurodegenerative disease, animal models

We all know that age-related neurodegenerative diseases affect aging individuals. So why does basic research continue to make use of the immature nervous system or mutants that succumb early and die young? And could this explain why strategies that rescue immature neurons fail to translate into effective clinical treatments for neurodegenerative diseases in aging humans? Here I try to make sense of this current state of affairs and suggest a pragmatic way forward.

The number of people over 60 years is expected to rise from 841 million in 2013 to more than 2 billion in 2050 (UN, 2013). As populations get older, age-related neurodegenerative diseases such as Alzheimer's Disease (AD) and Parkinson's Disease (PD) have become more common (Reitz et al., 2011; Reeve et al., 2014), and even for less common neurodegenerative diseases, such as Amyotrophic Lateral Sclerosis (ALS) this trend seems likely even, if it has not so far been proven (Beghi et al., 2006). Over the past 20–30 years we have witnessed much excitement following laboratory discoveries with the potential to translate into therapies for age-related neurodegenerative diseases (Oppenheim, 1996; Weissmiller and Wu, 2012), only to learn that these have failed in clinical trials (Glaser, 1997; Evans and Barker, 2008; Burns and Verfaillie, 2015), raising the question “what are we missing?” I suggest we are forgetting that age-related neurodegenerative diseases are just that: *age-related*. For AD, PD, and ALS, researchers have looked at everything from mis-folded proteins to infectious agents. As a result we now have acetyl cholinesterase inhibitors that transiently improve cognition in the early stages of AD (Bond et al., 2012), dopamine modifying drugs for the temporary amelioration of motor symptoms in the early stages of PD (Müller, 2012) and an NMDA antagonist which prolongs life for around 3 months in ALS (Gibson and Bromberg, 2012). However, none of these treatments based on studies of the immature nervous system alters the course of these age-related diseases. They remain incurable. Perhaps it is significant that while many animal models of age-related neurodegenerative diseases develop symptoms and die young (Gordon, 2013; Blesa and Przedborski, 2014; Neha et al., 2014), people with age-related neurodegenerative diseases develop symptoms when they are older and die when they are older. We already know that age at the time of neuronal injury affects neuronal survival (von Gudden, 1870; Lieberman, 1974; Aperghis et al., 2003), so it is a small step to go on to suggest that age-related differences in neuronal survival requirements could explain the disappointing translation of basic research to clinical situations. Some researchers may be unwilling to change the model that has been successfully funded for decades by the grant awarding bodies and perhaps *vice versa*. And therein lies the rub, because this will encourage hyperbole around basic science discoveries using immature systems and near silence when these discoveries fail to translate to aging humans. I suggest that one way out is to simply accept that if we want to know why neurons in the aging nervous system die, then we need more research on the aging nervous system. Of course, researching the aging nervous system is notoriously difficult. In most countries, aged animals cannot readily be obtained, and waiting for a colony to simply grow old is fraught with problems; research is put on hold for 2–3 years, age-related health problems such as kidney failure and lipomas impact on animal health and animals simply die of old age. Many of these age-related health problems, including an extension of lifespan, are avoided by using caloric-restricted animals, but how they relate to humans with an essential ad-libitum diet is unknown. Moreover, where it has been studied, caloric restriction impacts on neuronal survival in aged animals (Aperghis et al., 2003). For those with money to burn, aged rodents can be imported from the National Institutes on Aging in the USA. For a research in Australia, for example, this works out at around 40 times the cost of standard laboratory rodents with no guarantee the animals will survive the trip. And to make matters worse, unlike immature neurons where neurons die after all manner of perturbations (Greensmith and Vrbová, 1996; Blesa and Przedborski, 2014), it is difficult to get aged or adult neurons to die in experimental situations (Koliatsos et al., 1994; Mattsson et al., 1999). This clearly does not make for a good experimental model to test neuronal rescue if it is difficult to get the neurons to die in the first place. So there are clearly a number of challenges associated with the study of the aging nervous system to help us understand age-related neurodegenerative disease, but I maintain that these are not insurmountable obstacles. The costs of aging-related research can be reduced by a national collaborative efforts related to aging animal rearing and by the sharing of resources and information on those procedures that actually do cause aging neuronal loss. Only by national collaborative efforts will aging experimental models that more clearly address the clinical problem become available. And this is where political will comes in. The World Health Organisation (NIH/WHO, 2011) looked at 23 low-to middle- income nations and estimated that their combined loss in economic output between 2006 and 2015 due to age-related diseases was USD84 billion, and the global cost of AD alone in 2010 was estimated at USD604 billion (Wimo et al., 2013). Against this, the cost of using aging animals in research is tiny, so the economic case is solid. If we accept that we have ample evidence that the immature nervous system is an inappropriate model to develop therapeutic strategies for age-related neurodegenerative disease, then the obvious conclusion is to invest in research on the aging nervous system.

## Conflict of interest statement

The author declares that the research was conducted in the absence of any commercial or financial relationships that could be construed as a potential conflict of interest.
